# Diverse polysaccharide production and biofilm formation abilities of clinical Klebsiella pneumoniae

**DOI:** 10.21203/rs.3.rs-4630973/v1

**Published:** 2024-08-09

**Authors:** Renee Fleeman, Robert Beckman, Elenora Cella, Taj Azarian, Olaya Rendueles

**Affiliations:** University of Central Florida; University of Central Florida; University of Central Florida; University of Central Florida; Center of Integrative Biology

## Abstract

*Klebsiella pneumoniae* infections have become a growing threat for human health. The lack of understanding of the relationship between antibiotic resistance, mucoviscosity, and biofilm formation in clinical isolates impedes our abilities to effectively predict K. *pneumoniae* infection outcomes. These traits are also associated with fitness in natural populations and more specifically within a host. The Multidrug-Resistant Organism Repository and Surveillance Network offers a unique opportunity into the genetic and phenotypic variabilities in the *K. pneumoniae* isolates encountered in the clinics today. To this end, we compared the genetic profiles of these isolates with the phenotypic biofilm formation abilities, percent mucoviscosity, and growth rates. We found most isolates formed limited biofilm, although a select group of isolates could form extremely robust biofilms. Variation in biofilm formation could not be explained by difference in growth rate, suggesting specific genetic and physical determinants. Interestingly, the most mucoid strains in the populations were lacking the genetic element regulating the mucoid phenotype and three of these isolates were able to form robust biofilms. There was a significant phenotype-genotype correlation with decreased biofilm formation and an insertion sequence in the transcriptional activator of the type III fimbrial system. Finally, confocal microscopy highlighted the structural and spatial heterogeneity of biofilm among the most robust biofilm formers not detected by traditional methods. The combination of phenotypic, genomic and image analyses allowed us to reveal an unexpected phenotypic diversity and an intricate relation between growth, mucoviscosity and specific virulence-associated genetic determinants.

## Introduction

*Klebsiella pneumoniae* is an emerging threat to human health due to its extreme rate of drug resistance acquisition^1–3^. Until recently, these strains have primarily infected immune compromised patients due to their lack of virulence factors to bypass the host immune system^4 5^. However, horizontal transfer of large virulence plasmids within the K. *pneumoniae* species has now allowed for hypervirulent isolates to emerge within the community and infect healthy individuals^6–8^. Historically, the hypermucoviscous phenotype was primarily observed with *K. pneumoniae* hypervirulent K1 and K2 capsule serotypes^9^ and their increased mucoviscosity has been shown to be due to the virulence plasmid pLVPK encoding genes for production of RmpA, the transcriptional regulator of the mucoid phenotype^10^. However, recent studies have shown the spread of this virulence plasmid to other capsule serotypes^11^. Virulence acquisition in combination with the pan drug resistance status of many isolates of *K. pneumoniae* has elevated this species to the CDC urgent threats for concern to human health^12^. Interestingly, difficulties have been encountered accurately predicting virulence in clinical K. *pneumoniae*^13 14^.

Surface attachment and biofilm formation are a crucial first step of the infection process, but this important virulence trait is difficult to link to a genotype, or predict from genomic data,^15–17^. Characterization of the matrix of *K. pneumoniaebio* films found it is largely composed of lipopolysaccharides and capsular polysaccharides^18, 19^. Previous studies have highlighted the importance of capsular polysaccharides in biofilm formation and implicated its deleterious effect through occluding the fimbriae that allow for surface attachment^20, 21^. The capsule has been shown to have a positive or negative correlation to biofilm formation depending on the overall ability of the isolate to form biofilms^22,23^. Furthermore, the deletion of capsule in a hypermucoviscous K1 serotype isolate increases biofilm formation abilities but decreases biofilm formation with a K124 serotype isolate, indicating adhesion factors and capsule abundance together drive biofilm formation^23^. These findings have highlighted the need to better understand how highly capsulated *K. pneumoniae* can bypass the impediment caused by capsule to form robust biofilms. *K. pneumoniae* attachment is mediated by the chaperone-usher systems type I and type III fimbriae encoded by the *fimA-K* and *mrkA-I* operons, respectively^22, 24^. Mutations within *mrkD* and *fimH*, which are the tip adhesins of each fimbrial system, have been shown to vary based on the characteristics of the isolate that acquired the mutation^22^.

Here we determine biofilm formation potential of the Multidrug-Resistant Organism Repository and Surveillance Network (MRSN)^25^. The MRSN collection is a diverse set of *K. pneumoniae* covering a broad range of the potential multi-locus sequence type (MLST) with the genome sequencing data and curated metadata available for all isolates. Therefore, this collection is optimal to compare the biofilm formation characteristics observed to the genetic profiles for a greater understanding the range of physical attributes important for strong biofilm formation. To do so, we leveraged classical phenotypic tests with comparative genomics and confocal microscopy. We assessed biofilm formation, biofilm structure/morphology, mucoviscosity, and growth rate of the MRSN collection in order to align our results to their phylogeny and fimbriae allele variations. For deeper insight, we enriched the dataset with the virulence and antibiotic resistance scores from the original publication of this set, along with the host isolation source^25^. Our analyses identified a surprising diversity in the ability to form biofilms, encompassing both mucoid isolates and non-mucoid isolates. Additionally, we show only two isolates displaying increased mucoviscosity carry the *rmpA* genetic element encoding for the hypermucoviscous phenotype^9^, suggesting that other genetic mechanisms can determine mucoviscosity in a broader set of isolates. In line with previous studies, we identified mutations within the tip-adhesin of type 3 fimbriae, encoded by *mrkD*, were most prevalent within the mucoid isolates and insertion sequences in *mrkH were* strongly correlated with decreased biofilm formation. Finally, our confocal imaging analysis reveals matrix composition variability between mucoid and non-mucoid isolates that form robust biofilms. Our work reveals the presence of a mucoid phenotype without the genetic elements can be aligned with specific fimbriae mutations and the composition of the matrix varies greatly depending on the mucoviscosity of the isolates.

## Results

### Biofilm formation is greatest with urine isolates and occurs in both classical and hypervirulent pathotypes

**Figures 1:**
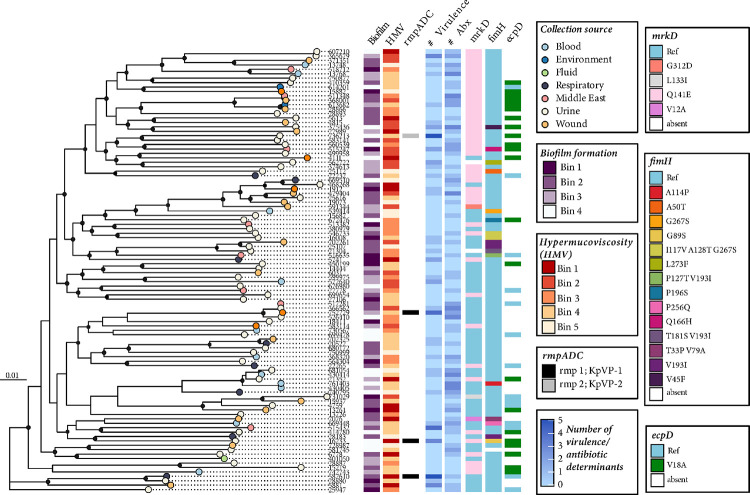
Genome alignment of the MRSN collection with the biofilm and mucoviscosity phenotypic analyses. The figure shows the maximum likelihood phylogeny illustrating population structure, phenotypic biofilm formation, phenotypic mucoviscosity, genotypic mucoid regulator presence *(rmpADC)*, genotypic virulence and antibiotic resistance determinants, and typing of *mrKD, fimH* and *ecpD* genes among *K. pneumoniae* isolates (N=100) previously published^25^. Tip shades indicate the host isolation source from the original publication^25^. A black circle along the branch indicated a higher bootstrap values (> 0.7). The presence of determinants is shown with a colored rectangle on the heatmap according to the legend on the right.

To rigorously test biofilm formation across the 100 diverse *K. pneumoniae* isolates of the MRSN collection, we grew all strains statically in TSB supplemented with 0.5% glucose, a robust medium providing the most optimal biofilm formation conditions for our analysis. We then used a crystal violet staining readout and measured optical density at 550 nm to determine biofilm density without bias for cells or polysaccharide, as this dye stains both of these elements^26^. The phylogeny was inferred from a core-genome alignment of 3,729 Clusters of Orthologous Groups (COGs) of proteins and 149,624 single nucleotide polymorphism (SNP) sites (Fig. 1). This resulted in several well-supported clades, representing a broad range of MLSTs, which is in line with what was observed in the original work with these isolates^25^. When comparing the biofilm formation of the 100 isolates, we saw a great diversity of biofilm formation abilities, ranging from no visible biofilm to formation of a very robust biofilm. Such heterogeneity indicates that biofilm is one of the most diverse and versatile traits in *K. pneumoniae*, as revealed by the high diversity index (Table 1). This is also observable in the distribution of biofilm formation, which showcases a long tail composed of *ca*. 15% of strains with an exceptionally strong capacity to form biofilm (**Supplemental Figure S1A**). Furthermore, the diversity of biofilm formation was spread between the clades, with each clade composed of both strong and weak biofilm formers, suggesting skim influence of phylogeny (Table 2). Our analysis was deposited to be made publicly available and consultable (https://microreact.org/).

**Figure 2:**
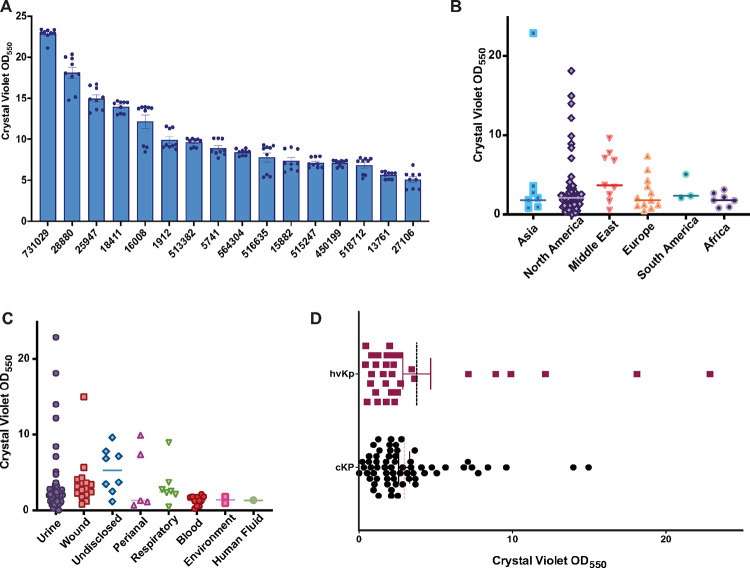
Biofilm formation is greatest with urine isolates and occurs in both classical and hypervirulent pathotypes. The figures show the biofilm density of the isolates as a OD_550_ readout. Figure 2A and 2B show the respective geographical location and host isolation site of the isolates. Figure 2C shows the biofilm density of cKp (virulence score 0) and the hvKp (virulence score 1–5) with a line a OD_550_ as the selection cutoff for the strongest biofilm formers. Figure 2D is the 15 strongest biofilm formers biofilm density next to their growth on both congo red agar and calcofluor white agar. Figures 2A, 2B, and 2C show the mean density of each isolate with the mean of the population as a dotted line. Figure 2D shows the mean of n=9 biofilms for each isolate with error as SEM.

We found overall many isolates in the diversity set had limited biofilm formation, with 38 isolates displaying moderate biofilm formation (**Supplemental Figure S1B**), and 46 isolates formed very limited biofilm (**Supplemental Figure S1C**). However, a select group of 16 isolates formed extremely dense biofilms (bin 1 crystal violet OD_550_ > 5) (Fig. 2A). To begin our comparison of the covariates which may influence biofilm formation, we first assessed the geography, patient isolation, and pathotype. When comparing the geographical origin with the biofilm formation abilities, as expected we did not find any correlation. Of note, we found that a sample isolated from Asia (MRSN 731029) had the greatest biofilm formation of the diversity set, samples from North America exhibited the most diverse formation, and samples from the Middle East, on average, formed the most robust biofilms (Fig. 2B). When considering the host isolation site, samples isolated from patient urine and wounds had the most diversity of biofilm formation abilities including those with the greatest biofilm formation, while isolates from the blood, human fluid, and the environment had the least ability to form biofilms (Fig. 2C). To observe the variability of biofilm formation with the presence of virulence genes, we graphed the *K. pneumoniae* isolates with virulence scores 1–5 (hvKp) next to those with a virulence score of 0 (cKp)^25^. Although we found the mean formation abilities were equal, the two isolates with the greatest biofilm formation abilities were hvKp isolates (Fig. 2D).

Since biofilm formation abilities have been previously linked to curli fimbriae and cellulose production^27, 28^, we assessed the abilities of the MRSN collection for production of these factors as previously described using congo red agar and calcofluor white agar plates, respectively^29^. From our congo red analysis, we found a great diversity between the 100 isolates within the collection (**Supplemental Fig. 2**). Specifically, MRSN 564304 produced dark red phenotype indicative of curli production, while the strongest biofilm former MRSN 731029 displayed very little color. Furthermore, MRSN isolates 1912 and 5741 displayed a pink color rather than red possibly due to the abundance of capsule production like what is observed with the control hypervirulent *K. pneumoniae* NTUH (**Supplemental Fig. 2**). However, similar to what was recently shown with *Klebsiella variicola*^30^, within the entire collection, the operon *csgABCDEF was* not present suggesting the phenotypes observed on congo red is due to another factor. In complementation of the congo red analysis, when grown on calcofluor white agar and visualized under UV fluorescence we saw diverse fluorescence, indicating variability in cellulose production within the entire collection (**Supplemental Figure S2**). As opposed to the curli operon, we did find the *bcsABCDEFGQZ* operon encoding for production of cellulose within the genomes of the collection. Most isolates had a complete operon, with exception of MRSN 736213, 16008, and 450199 that had *bcsABCZ, bcsBCZ*, and *bcsABCEFQZ*, respectively. Although MRSN 736213 had limited biofilm formation, MRSN 16008 and 450199 were strong biofilm formers. Collectively, we conclude that the strongest biofilm formers were isolated from the urine, both cKP and hvKp are capable of forming robust biofilms, and the presence of curli and cellulose are not sufficient to describe the biofilm formation abilities of this collection suggesting other factors are contributing to biofilm formation.

### Mucoviscosity limits biofilm formation and is not exclusive to the K1 and K2 capsule serotype

Literature to date has shown that capsule is important for biofilm formation^31, 32^. However, the increased encapsulation has been suggested to interfere with the attachment of bacterial cells resulting in decreased biofilm formation^21^. To investigate the correlation between mucoviscosity and biofilm formation in this diverse set of *K. pneumoniae* isolates, we assessed the percent mucoviscosity to compare to our biofilm density analysis. This assessment was done as previously described using slow centrifugation and calculating the percent mucoviscosity as the change in supernatant optical density at 600 nm^14 19 33^.

**Figure 3:**
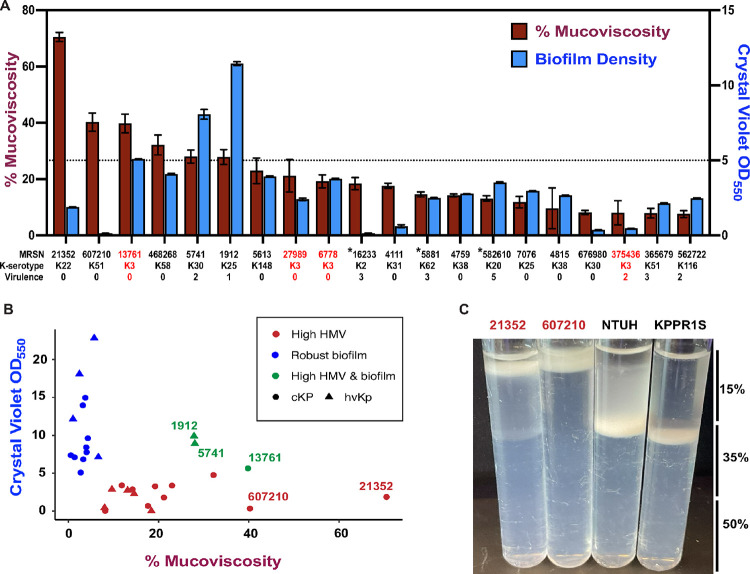
Mucoviscosity limits biofilm formation and is not exclusive to the K1 and K2 capsule serotype. The figures show percent mucoviscosity compared to biofilm density and the density of the most mucoid isolates in the collection compared to hypermucoviscous isolates. Figure 3A shows the 20 isolates with % mucoviscosity >10% on the left y-axis and the biofilm density on the right y-axis. The K serotype of each isolate is show along with the virulence score. The isolates that have the *rmpA* genetic element are marked with an asterisk and K3 capsule serotype are shown with red text. Error is shown as ± SEM. Figure 3B shows the percent mucoviscosity (x-axis) and biofilm formation (y-axis) of the top 15% biofilm formers and the top 15% mucoid isolates (HMV). The isolates that are mucoid and form robust biofilms are colored separately for comparison. Figure 3C shows the percoll density gradient migration of the two most mucoid isolates in the collection compared to the control hypermucoviscous isolates NTUH K2044 (K1) and KPPR1S (K2).

We found that most strains did not display high percent mucoviscosity, yet *ca*. 15% of strains exhibited important mucoviscosity (**Supplemental Figure S3A**). Such long tail in the distribution was similar to that of biofilm formation (**Supplemental Figure S1A**). Therefore, we assessed correlations between percent mucoviscosity and biofilm formation. First, we verified whether phylogeny strongly bias the analyses. We calculated both Pagel’s λ and Bloombergs K. We did not detect a significant phylogenetic signal in mucoviscosity and biofilm formation (Table 2). This could be explained by extensive horizontal gene transfer of virulence factors in these species, including the above mentioned *rmp* locus encoded in the hypervirulence plasmid^10^. Overall, there was not a significant correlation with mucoviscosity and biofilm formation when assessing all isolates (**Supplemental Figure S4B**), or when considering virulence as a covariant independently (**Supplemental Figure S4C and S4D**). We found that although K1 and K2 capsule serotypes are often associated with increased mucoviscosity^9^, among the 20% most mucoid isolates, only one had a K2 capsule serotype (Fig. 3A). Surprisingly, all isolates with a K3 capsule (N = 4) were among the most viscous, and significantly so (X^2^ = 11.8, P < 0.001). Furthermore, only three isolates (indicated with asterisk) in this set of isolates have the *rmpACD* genetic element shown to regulate the hypermucoviscous phenotype^34^. The analysis of the isolates with highest percent mucoviscosity and most robust biofilm formers suggest a trade-off between the two traits, with only three isolates displaying increased percent mucoviscosity being able to form high biofilm (Fig. 3B).

For a robust analysis, we included another readout of encapsulation and analyzed the isolates migration on a percoll density gradient^35^. This approach was selected over the more traditional uronic acid quantification due to the large diversity of uronic acid content of each serotype, making difficult comparison across serotypes. Our results were in line with the percent mucoviscosity results as we observed none of the most mucoid migrated to the bottom of the percoll gradient, indicating high capsule production in this set of isolates; while the least mucoid isolates had the most isolates that migrated to the bottom of the gradient, indicating low levels of encapsulation (**Supplemental Figure S4**). Surprisingly, the two most mucoid isolates MRSN 21352 and 607210 migrated less than the respective K1 and K2 capsule serotype hypermucoviscous controls *K. pneumoniae* NTUH K2044 and KPPR1S (Fig. 3C). Together, our results suggest although mucoviscosity limits biofilm formation but highly mucoid strains can form robust biofilms.

### Mucoviscosity and antimicrobial resistance impact growth rate

Previous work has highlighted the impact of population yield on biofilm formation^22^. To test if these findings applied more broadly than previously reported, and if like what was observed with our percent mucoviscosity and biofilm comparison, subsets of this collection had variable correlation with growth yield and mucoviscosity or biofilm. Therefore, we performed growth curve analyses with the isolates from the MRSN diversity panel to determine the generation time, maximum yield, and area under the curve (AUC). These parameters were then compared to the antibiotic resistance profile, virulence score, biofilm density, and percent mucoviscosity. Interestingly, we found that the growth rate parameters were much less diverse across the isolates than biofilm formation abilities (Table 1).

**Figure 4:**
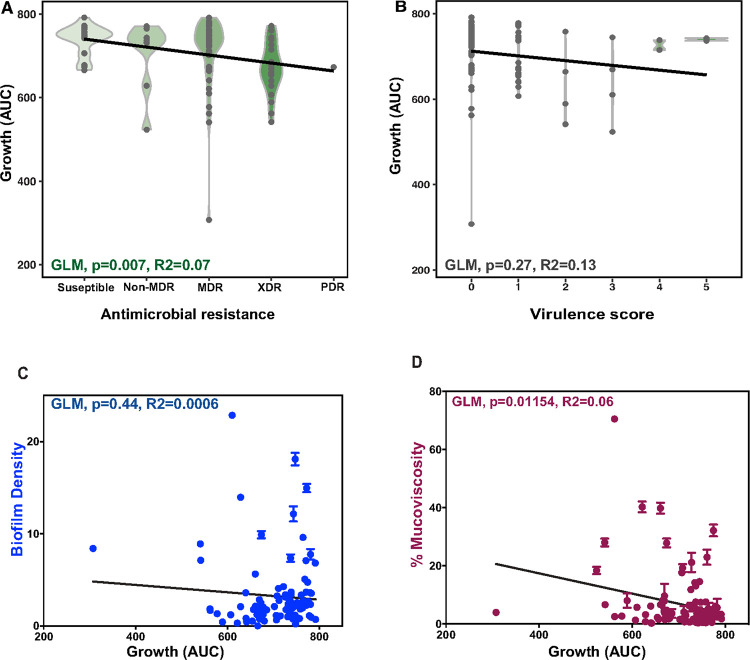
Mucoviscosity and antimicrobial resistance impact growth rate. The figures show area under the curve (AUC) of the growth rate analyses of the collection compared to covarities. Figures 4A and 4B show the AUC for isolates with different antimicrobial resistance levels (MDR, multiple drug resistance; XDR, extensive drug resistance; and PDR, pan drug resistance) and different virulence scores (0–5), respectively. Figures 4C and 4D show the AUC compared to biofilm density and percent mucoviscosity, respectively. General linear model was used to assess p value and R^2^ values shown.

As expected, we observe a correlation between mucoviscosity and virulence score (GLM, p value = < 0.0001). Overall, we saw a negative correlation with all parameters tested when compared to AUC (Fig. 4). Specifically, when comparing the drug resistance status with the growth curve analysis, we found a significant (GLM, p value = 0.007; R^2^ = 0.07) negative correlation with drug resistance acquisition AUC (Fig. 4A). There was also a significant (GLM, p value = 0.002; R^2^ = 0.09) positive correlation with generation time and drug resistance and a negative correlation (GLM,, p value = 0.009, R^2^ = 0.06) with the maximum yield and drug resistance (**Supplemental S6**). Although the multi-drug resistant (MDR) group had quite a diversity of growth yields, this was comparably less diverse than the biofilm formation and mucoviscosity of this subset of isolates. Similar to antibiotic resistance, virulence factors such as mucoviscosity have been reported to be costly to the growth rate^30^. Although the overall correlation was not as significant (Fig. 4B), we found that classical and hypervirulent strains did have differences in growth rate, as estimated by the area under the curve (AUC) (GLM, p value = 0.02; R^2^ = 0.03). In addition, there was a positive correlation generation time (GLM, p value = 0.061; R^2^ = 0.18) (**Supplemental Figure S6**).

Next, we compared our growth analyses to the biofilm formation capabilities and percent mucoviscosity, as previous work has shown that biofilm formation can be impacted by the growth rate of the population^12^ We found a negative correlation between AUC and both biofilm formation (GLM, p value = 0.44; R^2^ = 0.0006) (Fig. 4C) and percent mucoviscosity (GLM, p value = 0.01154, R^2^ = 0.06) (Fig. 4D). Interestingly, MRSN 564304 had a substantial growth defect when compared to representative strains, fast growers, the most mucoid strain, and the greatest biofilm former (**Supplemental Figure S7**). With the concern that this outlier with an extremely slow growth rate may be skewing the significance of the correlation considering the sensitivity of Pearson’s correlations, we removed it from the analyses. This resulted in no qualitative difference (GLM, p value = 0.001, R^2^ = 0.1). However, this strain was within our set of strong biofilm formers and was the one isolate that appeared dark red on congo red agar and dark blue on calcofluor white agar (Fig. 1D). These data suggest that mucoviscosity may affect the growth rate of the isolates, but growth rate does not affect biofilm formation.

### Fimbriae mutations differential impact on biofilm formation and mucoviscosity

It has recently been discovered that mutations within the gene encoding for the tip-adhesin of type III fimbriae *(mrkD)* or within the switch of type I fimbriae *(fimH)* impact biofilm formation in a capsule dependent manner^22^. Furthermore, a recent study revealed insertional inactivation of *mrkH*, encoding for a c-di-GMP transcriptional activator, resulted in decreased biofilm formation^30, 36^. In addition, another chaperon-usher system tip adhesin EcpD has been shown to be important for adherence to epithelial cells^37^. As expected, we found that biofilm and mucoviscosity does not correlate with genome size (Spearman, p value = 0.89 and 0.09, rho = 0.01 and − 0.16 for biofilm and mucoviscosity, respectively). Therefore, to capitalize on the diversity of sequenced isolates within this collection, we next aimed to assess the fimbriae allele variations to determine impacts on biofilm.

**Figure 5:**
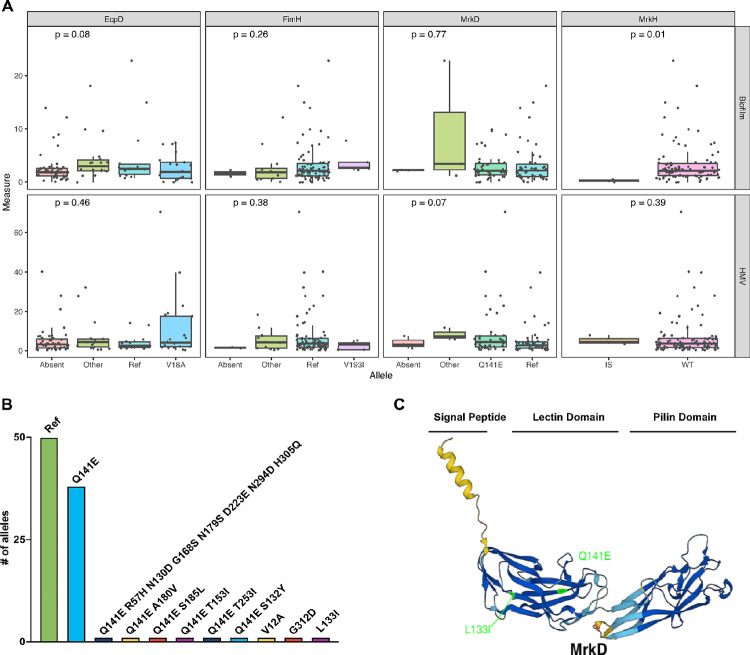
Fimbriae mutations differential impact on biofilm formation. The figures show the allele variations within the *mrkD* gene and their phenotypic biofilm, mucoviscosity, and growth rates. Figure 5A shows the fimbrea allele variations and their impact on biofilm formation and mucoviscosity. Kruskal-Wallis statistical anlaysis was used to determine the p values shown. Figure 5B shows the # of isolates within the collection that have the labeled mutation shown. Figure 5C is the alpha fold generated structure of the MrkD protein with the signal peptide, lectin domain, and pilin domain labeled. The Q141E and L133I mutations are shown in bright green.

We compared the *mrkH, mrkD, fimH, and ecpD* operons to identify variations from the most common allele (hereafter names as ‘reference’) and found a variety of alleles within the collection as well as isolates with the genetic elements absent from the genome (Fig. 1). Overall, there was a clear genotype-phenotype correlation with the three isolates (MRSN 560539, 375436, and 730567) that have insertion sequences in the *mrkH* gene and are all deficient in biofilm formation (Kruskal-Wallis, p value = 0.01) (Fig. 5A). In addition, there were many isolates with allelic variation in *mrkD* (10 different alleles with the dominant mutation being Q141E, present in 44 different isolates). Indeed, this mutation emerged early in the life history of *K. pneumoniae* and is present in most isolates from the first clade (Fig. 1). Interestingly, *mrkD* allelic variations did not show a correlation with biofilm changes but did have a minor impact on mucoviscosity (Kruskal-Wallis, p value = 0.07). However, the importance of this fimbriae system for biofilm formation is revealed by the two strains lacking either a portion or all of the *mrk* operon (MRSN 562722 and 21304, respectively) being among the lowest biofilm formers (Fig. 1 and **Supplemental Figure S1**). We observed a larger diversity of FimH alleles, a total of 17 different sequences, yet the reference was by far the most common. The second most common allele differed in V193I compared to the reference and was only present in three different isolates. No significant change in biofilm or mucoviscosity was seen with these mutations. Similar to the isolates with larger mutations of the *mrk* operon, we saw limited biofilm formation with MRSN 581745 that has an 829 base pair deletion between *fimG* and *fimH* (**Supplemental Figure S1**). Finally, we did not identify a homolog of *ecpD* tip adhesin gene in almost half of the isolates (and the most common change was observed in the signal peptide, V18A), (Fig. 5A). Interestingly, even though there was no overall impact (Kruskal-Wallis, p value = 0.46) of allele variations in the *ecpD* gene on mucoviscosity, those isolates with the V18A mutation had a wide range of mucoviscosity.

We found it extremely compelling that the Q141E allelic variation was found in 38 isolates as a single mutation and in six isolates with additional non-synonymous mutations in *mrkD* (Fig. 5B), many of which clustered together on the phylogenetic tree (Fig. 1). The Q141E mutation is located within the lectin binding domain of MrkD, shown to be important for binding affinity (Fig. 5C)^22^. Interestingly, the strongest biofilm former of the collection (MRSN 731029 collected from a urine sample in Asia) had a L133I mutation on the opposite side of the lectin binding domain (Fig. 5C). Comparatively, the mutations in *fimH* and *ecpD* occurred not in the lectin binding region but in the signal peptide and pilin domain, respectively (**Supplemental Figure S8A** and **S8B**). Our observations suggest mutations within the tip adhesion can have differential effects on the isolate’s biofilm formation and mucoviscosity but there is a strong correlation with a loss of biofilm formation associated with the *mrkH* insertion sequences disabling the c-di-GMP activator of the type III fimbriae.

### Environmental sheer flow influences the biofilm formation potential and spatial distribution in strong biofilm formers

With the diversity of attributes of the top biofilm formers we wanted to visualize their biofilm compositions to learn more about biofilm structure and potential heterogeneity in morphology. The biofilms of the highest biofilm formers (Fig. 2D) were grown in static conditions with the same growth media to mirror our crystal violet staining and in microfluidic conditions to understand the role of mucoviscosity and fimbriae mutations with different environmental sheer flow. To introduce environmental sheer flow, we grew them under flow rate of 65 μL hr^−1^ for 24-hours in a microfluidic 24-well plate that has a confocal microscopy compatible glass bottoms under the channels. Due to the increased serpentine clogging encountered when growing our *K. pneumoniae* isolates in the microfluidic plate using the TSB with 0.5% glucose we used M9 minimal media with 0.4% glucose as previously described for *Escherichia coli*^38, 39^. With both biofilm growth conditions, the bacterial population and polysaccharide matrix was stained with Syto9 and calcofluor white, respectively. Confocal z-stack imaging facilitated the 3D rendering images to visualize the height and composition of the biofilms formed by each isolate.

**Figure 6:**
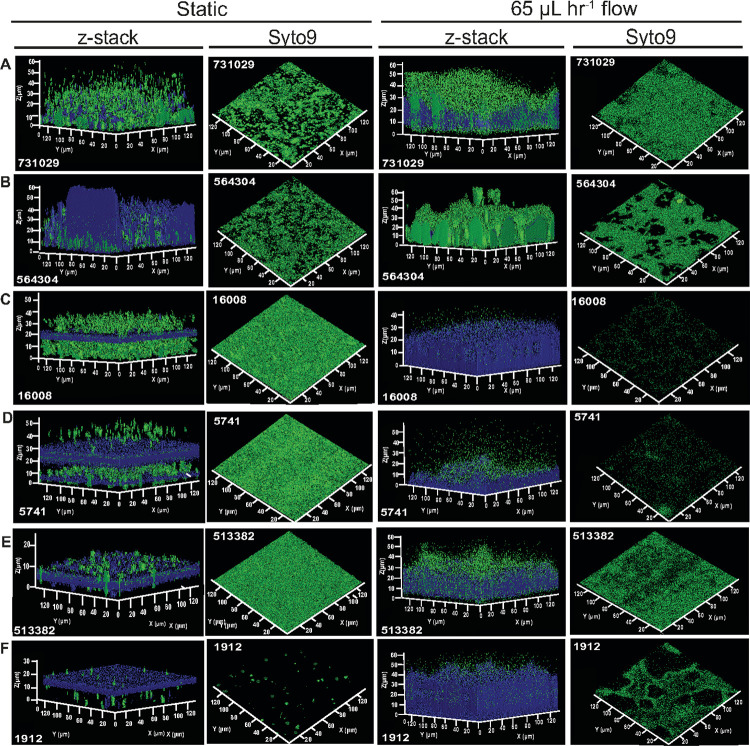
Environmental sheer flow influences the biofilm formation potential and spatial distribution in strong biofilm formers. The figures show the 3D rendering of confocal z-stack imaging of the 6 of the top biofilm forming isolates stained with Syto9 and calcofluor white to visualize the cells and polysaccharide matrix, respectively. Figures 6A – 6F show MRSN 731029, 564304, 16008, 5741, 513382, and 1912 biofilms, respectively. Each isolate is shown grown under static conditions and with 65 μl hr^−1^ sheer flow. For each isolate, with both conditions the z-stack images are shown to visualize the height of the biofilm and images from the top of the biofilm are shown to visualize the overall cellular density. n=3 biofilms were grown and imaged with a representative image shown.

We chose crystal violet staining as our measure for the MRSN diversity set biofilm formation because it is a robust, well studied method for high-throughput biofilm assessments; yet, there is no distinction between the cells and the matrix materials because crystal violet stains both without bias^37^. Therefore, considering the diversity of the phenotypes of our 15 top biofilm forming isolates we wanted to visualize the bacterial cells independently from the matrix polysaccharides and determine how these components change when the biofilms are grown under environmental sheer flow. We found that in both static and microfluidic conditions, all isolates formed biofilms with average height between 30–50 μm and were found to have some level of polysaccharides within their matrix (Fig. 6 and **Supplemental Figure S9**). Specifically, when grown in static conditions, MRSN 731029 and 564304 displayed the most biofilm height (~ 50 μm), although 564304 had more polysaccharide matrix (Fig. 6A and 6B). These isolates when viewed from the top of the matrix revealed the cellular population as aggregates in microcolonies, compared to MRSN 16008, 5741, and 513382 that had uniformly dispersed cellular population when viewed through the z-stacks. (Fig. 6C, 6D, and 6E). Interestingly, MRSN 16008 had a thin layer of polysaccharide matrix with an abundance of cells above and below but the mucoid isolate MRSN 5741 had two distinct layers of polysaccharide matrix with less cells outside of the matrix. MRSN 513382 had a robust layer of polysaccharide matrix but decreased matrix height compared to the other isolates within the top biofilm formers (Figs. 6E). The cellular staining of MRSN 1912 with Syto9 was limited and we hypothesized this was due to the thickness of the matrix layer. To test this, we visualized biofilms grown with MRSN 1912 harboring pSL6_RFP to allow for constitutive RFP expression. We saw a slight increase in the cellular population under the matrix cap, although this was still minimal compared to the other isolates (**Supplemental Figure S10**).

When testing the effect of sheer force on biofilm formation of the isolates we found both the microcolony formation (Fig. 6A and 6B) and the polysaccharide cap phenotypes were no longer present (Fig. 6C–6F). Strikingly, MRSN 731029 and 564304 still had the most cellular density, although the MRSN 564304 displayed large gaps in the matrix when viewed from the top. MRSN 16008 had an abundant amount of polysaccharide matrix but less cellular density compared to MRSN 731029 and 564304. In addition, the two mucoid isolates that were able to form robust biofilms from our crystal violet readout responded to sheer force differently. MRSN 5741 had much less biofilm height and cellular staining when grown under sheer flow (Fig. 6D), but MRSN 1912 had increased biofilm height and cellular staining (Fig. 6F). These results reveal the diversity of spatial distribution between strong biofilm formers and the impact of sheer flow has differential effects on mucoid isolates that form robust biofilms.

## Discussion

The MRSN diversity panel is a set of 100 *K. pneumoniae* isolates that include a broad spectrum of the MLST identified^25^. With such a diverse large set of isolates, investigating the covariates important for biofilm formation has potential to expand our current knowledge of *K. pneumoniae* biofilm formation abilities. The diversity and versatility of the *K*. *pneumoniae* species could preclude predictive analyses. Our findings suggest an important phenotypic diversity in this set of isolates and revealed an intricate relationship between growth, mucoviscosity, and specific genetic determinants. Furthermore, the large diversity revealed here raises questions concerning: 1) the relative roles of natural selection outside a host or during gut commensalism, 2) potential alteration of mutation rates during infection, and 3) how these factors impact the capacity *K. pneumoniae* to persist.

We found that the life history or origin of the isolates allowed for the selection of biofilm formation based on the structural organization of the environment. For example, there was a diverse range of biofilm formation abilities within the isolates collected from the urine, including the strongest biofilm former of the entire collection (Fig. 2B). This reflects the importance of the selection pressure for biofilm formation for bacteria in this environment, because establishing a urinary tract infection is largely impacted by the ability to form biofilms both on indwelling devices and within the urinary tract^40^. Furthermore, isolates that came from very structured environments (i.e., wound) displayed strong biofilm formation and those came from less structured sample site (i.e., blood) had less biofilm formation. Therefore, our work suggests that the isolates were adapted during or prior to the infection to the environment where it was isolated. This finding builds upon the previous work that revealed the structure of the environment largely impacts population evolution^30^

Intriguingly, we found that percent mucoviscosity is not restricted to the K1/K2 serotype isolates nor exclusively associated to the presence of the *rmp* locus, the regulator of the mucoid phenotype in *K. pneumoniae* as 17 isolates were mucoid in the absence of such genetic element (Fig. 3A)^9^. There were three isolates (MRSN 16233 (K2); 582610 (K20); and 752729 (K64)) and 5 isolates (MRSN 5881 (K62); 16233 (K2); 582610 (K20); 736213 (K2); and 752729 (K64)) with the *rmpA1* and *rmpA2* genetic elements, respectively. None of these isolates were strong biofilm formers and only three of these isolates (MRSN 16233, 5881, and 582610) displayed high percent mucoviscosity (Fig. 3A, marked with asterisk). The observation of increased mucoviscosity in the absence of *rmpA1* and *rmpA2* suggests there are other genetic determinants of mucoviscosity in *K. pneumoniae*. We show that the top 15 isolates are not mucoid and most of the isolates with high percent mucoviscosity do not form robust biofilms (Fig. 3B). However, there are three outliers that are able to bypass the impediment mucoviscosity has on biofilm formation, suggesting these isolates can bypass the limitations of increased mucoviscosity on biofilm formation.

When looking at growth as a covariant, we found growth did not impact biofilm, as there was no significance correlation identified and one of the strongest biofilm formers was shown to have a noteworthy growth defect (Figs. 4C and **Supplemental Figure S7C**). When considering the other parameters of our study, we found that antibiotic resistance and mucoviscosity had a significant negative impact on the growth rate, but the virulence score was not associated with the growth rate (Fig. 4A, 4B, and 4D). These findings reveal the energetic burden antimicrobial resistance and increased mucoviscosity and suggests that the strict regulation of virulence traits can mitigate the effects on growth rate. This finding is important when considering the population dynamics suggesting that when removed from an environment where mucoviscosity or antibiotic resistance is not necessary, they may be outcompeted and less prone to spread a worrisome trait. When considering the fimbriae allele variants we found that, in line with recent work^30, 36^, insertion sequences in *mrkH* led to decreased biofilm formation, as all three isolates with this insertion displayed limited biofilm formation (Fig. 5). Interestingly, the mutations within *mrkD* had more of an effect on mucoviscosity than biofilm formation. Furthermore, the independent emergence of the Q141E allele throughout the life history of *K. pneumoniae* suggests it could provide a strong fitness advantage in the clinic. Alternatively, it could imply that it could be hitchhiking along several other mutational events. Conversely, *ecpD* allele variation was more impactful to biofilm (p value = 0.08) than mucoviscosity (p value = 0.46). Specifically, mutations within the tip adhesion showed increased biofilm formation, while a lack of an *ecpD* homolog within the genome led to a decrease in biofilm formation.

When visualizing the strongest biofilm formers using confocal microscopy we continued to see suggestions of adaptation to the environment. In particular, isolated from a respiratory sample and having high percent mucoviscosity MRSN 5741 displayed limited biofilm formation ability under 65 μL hr^−1^ sheer flow compared to static growth. Conversely, MRSN 1912 was collected from a perianal sample and although it had high percent mucoviscosity was able to form a more robust biofilm in the presence of environmental sheer flow, although the cellular population was not as dense as MRSN 731029 and 564303 isolated from urine samples. Although MRSN 564304 retained biofilm height with sheer flow, this isolate had a striking decrease in polysaccharide abundance, consistent with its dark phenotype when grown on congo red and calcofluor white agar (**Supplemental Figure S2**). This could be suggestive that the polysaccharide abundance observed in our static growth conditions are being washed away under sheer flow. The confocal analysis of the strong biofilm formers under environmental sheer flow not only suggests environmental adaptation but reveals the limitations of biofilm analyses using a single condition.

In conclusion, our investigation of the MRSN diversity panel has revealed a striking quantitative and morphological variation in the biofilm formation abilities within this set, reflecting the diversity of genetic components impacting biofilm growth. We found that mucoviscosity and antibiotic resistance largely impact the growth parameters of the isolate, while the tight regulation of virulence may offset effects on growth potential. Ultimately, *mkD* tip adhesin mutations resulted in increasing mucoviscosity at cost of biofilm formation and mutations in other tip adhesins may have a role in rescuing this deficit. Our work has significantly improved our knowledge of the covariates of biofilm formation and our understanding of the genetic variabilities of the attributes important for *K. pneumoniae*.

## Methods

### Crystal Violet Biofilm Staining

To assess for biofilm formation, the strains were cultured in lysogeny broth (LB) and placed in shaking incubator (5 × g) for 24 hours at 37 °C. The overnight cultures were diluted to an OD_600_ of 0.5 (9.75 × 10^9^) in biofilm media (tryptic soy broth and 0.5% glucose). The biofilms were grown in tissue culture treated 6-well plates in triplicate for 24 hours at 37 °C static. Supernatant was removed from wells and biofilms were washed with 1 mL phosphate-buffered saline (PBS). Biofilms were stained with 1 mL 0.1% Crystal Violet (CV) for 15 minutes and placed in a fume hood for 24 hours. De-staining of biofilms was performed by adding 1 mL 30% acetic acid to wells for 15 minutes and transferring the mixture to a new 96-well plate. Microplate reader was used to measure the optical density (OD) at 550 nm.

### Congo red and Calcofluor white agar plating

Overnight cultures were grown in lysogeny broth (LB) with shaking (5 × g) for 24 hours at 37 °C. Overnight cultures were diluted 1:100 with LB media in a 96-well plate. Congo Red plates were prepared by adding 4 mL 0.1% Congo Red solution and 3.6 mL 50% sucrose solution to LB agar for a final volume of 50 mL. 10 mL 200 μg mL^−1^ calcofluor white staining dye was added to 40 mL LB agar for a final volume of 50 mL. 5 μL diluted cultures were spot plated onto dried Congo Red and calcofluor white plates and imaged after 24 hours.

### Mucoviscosity Assay

To assess for the hypermucoviscous phenotype characteristic of hypervirulent *K. pneumoniae*, the strains were cultured in LB with shaking (220 rpm) for 24 hours at 37 °C. 1 mL of overnight cultures were placed in 1.5 mL microcentrifuge tubes and OD_600_ values were recorded before and after centrifugation at 1,000 X g for 5 min. Mucoviscosity was calculated as Percent $\frac{\left(\text{OD}600\text{nmaftercentrifuge}\right)}{\left(\text{OD}600\text{nmbeforecentrifuge}\right)}\times 100$. The resulting values were graphed as mean percent mucoviscosity with error reported as ±SEM.

### Percoll Gradient Assay

Percoll^™^ (GE Healthcare) solutions (15, 35 and 50%) were prepared by combining percoll solution with phosphate-buffered saline (PBS) in 50 mL conical tube to achieve 50%, 35%, and 15% concentrations. 2 mL of the 50% percoll solution was placed at the bottom of 15 mL centrifuge tube followed by 2 mL of 35% then 15% Percoll solution. Strains were cultured in LB with shaking (5 × g) for 24 hours at 37 °C. Overnight cultures were centrifuged at 1,807 × g for 10 minutes, the supernatant was removed, and the pellets were resuspended in 600 mL PBS. 400 mL of resuspended cultures were pipetted on top of percoll gradients and centrifuged at 3,000 × g (room temperature) for 30 minutes. All strains were tested in triplicate and representative photos were shown for each.

### Genome alignment and tree generation

Genome sequencing data was acquired from the authors of the original manuscript at Walter Reed Army Institute of Research (WRAIR)^25^. Raw sequencing reads were filtered using Trimmomatic v.0.39 and quality assessed using FastQC^41^. *De novo* genome assemblies were constructed using Unicycler v0.4.8^37^ and annotated using Prokka v1.14.15^42^. Pangenome analysis was performed using Roary v.3.12^43^ and a core genome SNP alignment was extracted using snp-sites v2.4.0. A maximum likelihood (ML) phylogeny was inferred with IQTREE v1.6.8 using the ASC + GTR + GAMMA substitution model with 100 bootstrap replicates and rooted using MRSN25947 strain (ST5447)^44^ The Kleborate tool^45^ was then used to perform K and O typing using the --kaptive option^46^

To descriptively assess the distribution of phenotypic and genotypic determinants, phenotypic biofilm formation, phenotypic hypermucoviscosity, genotypic hypermucoidy (*rmpADC*), genotypic virulence determinants, genotypic antibiotic resistance determinants, and allele typing of *mrKD, fimH*, and *ecpD* genes, were mapped onto the core-genome phylogeny using ggTree in RStudio, running R version 4.3.1^47^ We aligned the phylogeny of the isolates with their biofilm formation and mucoviscosity by grouping into bins to aid in visualization of the alignments (Biofilm formation bin 1 – bin 4; HMV bin 1 – bin 5). For biofilm formation grouping, we assigned a cutoff of OD_550_ greater than 5 for the strongest biofilm formers in bin 1; OD_550_ greater than 2 and 1 for moderate biofilm formers in bins 2 and 3, respectively; and OD_550_ lower than 1 for very weak biofilm formers in bin 4. For mucoviscosity grouping (HMV), we assigned a curoff of > 10% mucoviscosity for the most mucoid isolates in bin 1, between 5–10% mucoviscosity in bin 2, between 5–3% mucoviscosity in bin 3, between 3–1% mucoviscosity in bin 4, and 19 < 1% mucoviscosity in bin 5.

### Identification of tip adhesins, curli and cellulose operon

Identification of the cellulose operon and curli biogenesis apparatus was performed as described previously^30^. Briefly, the experimentally validated protein sequences involved in curli and cellulose synthesis and identified previously^30^ were used as a query. For the identification of tip adhesins, the following proteins were used as query WP_004149659.1, BAH65076.1, WP_002890060.1 for MrkD, FimH and EcpD, respectively. BlastP (v2.7.1 +) with default parameters was used to search for each protein in the proteome of the MRSN collection. (i) *Curli*. Hits for the regulatory protein CsgD were identified, but no hits were obtained (E-value < 10^−5^ & identity > 60%) for any other protein, indicative of the absence of curli synthesis. (ii) *Cellulose*. For each proteome, we search for hits in each protein of the *bcs* operon (E-value < 10^−5^ & identity > 60%). All hits in each proteome colocalized, and the operon structure was inferred. Ninety seven out of the 100 genomes had a genomic architecture corresponding to *bcsGFEQABZC*. (iii) *Tip adhesins*. Sequences with an identity percentage of less than 80% were discarded. Reducing the threshold to 70% or increasing it to 90% did not alter the number of sequences discarded. All genomes had either one or zero hits per adhesin. To identify the different alleles, the most common protein sequence was labelled as reference. All other proteins were aligned to the reference using the *pairwiseAlignment* function from Biostrings and differences were identified with *mismatchTable* function. The AlphaFold Protein Structure Database was used to predict and generate the fimbriae structures, last accessed on March 2024^48, 49^.

### Growth curve parameters

Overnight cultures were diluted at 1 : 100 in fresh LB medium. Two hundred microliters of each subculture was transferred into 96-well microplate. Absorbance (OD_600_) of cell cultures was measured with a TECAN Genios^™^ plate reader. Absorbance values from within-block technical replicates were averaged and these averages were used as statistically independent data points. (*i*) *Growth rate*. Minimum generation times were estimated across replicates for the 1 h interval (ΔT) spanning the fastest growth during the exponential growth phase. This was calculated as follows:

$$\frac{1}{Td}=\frac{{\text{log}}_{10}\left({\text{maximum}}_{\text{OD}600}/{\text{minimum}}_{\text{OD}600}\right)}{\left({\text{log}}_{10}\right)\left(2\right)*\Delta T}\times 100$$

(ii) *Maximum yield*. This measure corresponds to the maximal OD_600_ reached by each culture. (*iii*) *Area under the curve* (AUC). AUC takes into account the lag phase, growth rate and population yield of the culture. It was calculated using the R function *trapz* from the *pracma* package.

### Biofilm formation static growth for confocal imaging

Overnight cultures were grown in LB in shaking incubator (220 rpm) at 37 °C for 24 hours. Overnight cultures were standardized to OD_600_ 0.5 (9.75 × 10^9^) in biofilm media (TSB media with 0.5% glucose). 1 mL of OD_600_ 0.5 (9.75 × 10^9^) standardized cultures were pipetted into Matsunami Glass bottom dishes (Glass Diameter: 14 mm, Glass Thickness: #1.5 (0.16–0.19 mm). Matsunami dishes were parafilmed and placed in 37 °C static incubator for 24 hours. After 24-hour growth, supernatant of biofilms was removed, and samples were washed with 1 mL 1X PBS. 1 mL of 5 μM SYTO 9 green-fluorescent stain diluted in 1X PBS was pipetted onto biofilm samples and rocked at medium speed for 1 hour. Dye was removed, and samples were washed with 1 mL 1X PBS. 1 mL of 50 μg mL^−1^ calcofluor white dye diluted in molecular grade water was pipetted onto all samples and rocked at medium speed for 5 minutes. Dye was removed, and samples were washed with 1 mL 1X PBS. Images were recorded using z-stack confocal microscopy imaging. For imaging of MRSN 1912 with constitutive RFP expression we electroporated pBTK1007 and post stained with calcofluor white stain. pBTK1007 (pSL6) was a gift from Jeffrey Barrick (Addgene plasmid # 191002; http://n2t.net/addgene:191002; RRID:Addgene_191002)^50^.

### Biofilm formation in microfluidic device

To introduce environmental sheer flow, we utilized the BioFlux One system (Cell Microsystems, Durham, NC; https://cellmicrosystems.com/bioflux/). This is an automated electropneumatic pumping system with associated operating software and a proprietary Well Plate Microfluidic^™^ device to increase the throughput of analysis of biofilm formation under physiological shear flow. The system is designed for flow control and accuracy, with automated pressure controllers that can control shear force to ± 0.05 dyn/cm^2^. The plates utilize a standard well plate format with an imaging surface having a #1.5 170 μm glass bottom and imaging channel dimensions of 350 μm wide, 70μm deep, and 4mm long. Using the BioFlux One device and a 48-well low shear plate (0–20 dyne/cm^2^), we tested the top biofilm formers from our crystal violet analysis. Briefly, overnight cultures were grown in LB in shaking incubator (220 rpm) at 37 °C for 24 hours. Overnight cultures were standardized to OD_600_ 0.5 (9.75 × 10^9^) in minimal media (with 0.4% glucose)^38^ pre-warmed to 37 °C. After warming the media, outlet wells received 40 μLsterile minimal media (with glucose), and flow was directed from outlet to inlet wells at 20 dyne/cm^2^ (2,356 μL hr^−1^) for 40 seconds. 100 μL of sterile media was immediately added to inlet wells to avoid drying. Residual media was removed from outlet wells, and 20 μL standardized cultures in pre-warmed media were added to outlet wells. Flow was directed from outlet to inlet wells at 4 dyne/cm^2^ (471 μL hr^−1^) for 5 seconds. 48-well low shear plate (0–20 dyne/cm^2^) was incubated for one hour at 37 °C. Outlet wells were cleared of all liquid and rinsed with 100 μL fresh sterile media (37 °C). 1 mL 37 °C fresh sterile media was added to inlet wells, and flow was directed from inlet to outlet wells at 1 dyne/cm^2^ (118 μL hr^−1^) for 5 minutes. Outlet wells were rinsed with 100 μL 37 °C fresh sterile media, and inlet wells were assessed to ensure 1 mL media was still present. 48-well low shear plate (0–20 dyne/cm^2^) received flow from inlet to outlet wells at 0.55 dyne/cm^2^ (65 μL hr^−1^) for 24 hours. Images of 48-well low shear plate (0–20 dyne/cm^2^) were recorded using Z-stack confocal microscopy imaging.

### Confocal microscopy z-stack imaging

Samples were transported in light protected blackout bin due to light sensitive staining dyes. Zeiss LSM 710 confocal microscope and ZEN microscope software were powered on. 63X oil immersion lens was used to image samples. Small droplet of lens oil was applied to microscope lens and the Matsunami Glass dish or the Bioflux 48-well low sheer plate containing sample was placed onto microscope lens holder. The microscope was lowered and GFP fluorescent detection laser was turned on and used to bring cells into focus. Once in focus, z-stack images were taken with using 488 and 543 nm laser channels for imaging of calcofluor white and Syto9 staining, respectively. The figures shown are 3D renderings of the z-stack images obtained to show biofilm thickness on the z-axis using a collective of images from multiple focal planes to produce a single 3D image.

### Statistics

Many statistics were performed with R v 4.3.1. (*i*) *Correlations between continuous variables*. To correlate the different continuous variables (growth parameters, HMV and biofilm) Spearman’s rank correlation was used. (ii) *Correlations between discrete and continuous variables*. We used general linear models to test the association between the different virulence, drug-resistance scores with mucoviscosity, and biofilm and other covariates using the *glm* function. We fitted the model with either resistance or virulence as dependent variables (Y) and HMV, Biofilm and growth were independent variables (X), following the formula Y~X. We assessed the relevance of the focal independent variable by testing if the parameter estimate for the variable was significantly different from zero (when the overall model had an R^2^ significantly higher than zero). (iii) *Estimate of phylogenetic inertia*. The presence of phylogenetic signal in the evolution of traits was estimated with Pagel’s lambda and Bloombergs K using the *phylosig* function of the *phytools* package v.2.1–1 for R^51^.

## Figures and Tables

**Table 1:** The diversity index weighing the variance of a trait, allowing direct comparisons across traits.

Trait	*I*_*x*_
Biofilm	1.18
Mucoviscosity	1.52
AUC	0.1
Generation time	0.1
Maximum yield	0.11

* Diversity indexes were calculated as the standard deviation of a sample, divided by the mean.

**Table 2:** Phylogenetic inertia calculated using Bloombergs K and Pagels λ. We estimated the phylogenetic inertia of all tested variables using Pagel’s λ and Bloombergs K with the function included in the *phytools* package and a phylogenetic tree based on the core genome. The null hypothesis is λ = 0 (no phylogenetic effect).

Trait	Bloomberg’s K	P value K	Pagels λ	P value Pagel
FimH	0.07	0.540	0.73	0.008
MrkD	0.58	0.008	1.00	0.012
EcpD	0.05	0.329	0.84	<0.001
AMR	0.13	0.127	0.73	<0.001
Virulence	0.05	0.665	0.00	1.000
AUC	0.13	0.210	0.71	0.003
Biofilm	0.30	0.063	0.00	1.000
HMV	0.06	0.650	0.00	1.000
